# Pulmonary Iron Homeostasis in Hepcidin Knockout Mice

**DOI:** 10.3389/fphys.2017.00804

**Published:** 2017-10-17

**Authors:** Jean-Christophe Deschemin, Jacques R. R. Mathieu, Sara Zumerle, Carole Peyssonnaux, Sophie Vaulont

**Affiliations:** ^1^Institut National de la Santé et de la Recherche Médicale, U1016 Institut Cochin, Paris, France; ^2^Centre National de la Recherche Scientifique, UMR 8104, Paris, France; ^3^Université Paris Descartes, Sorbonne Paris Cité, Paris, France; ^4^Laboratory of Excellence GR-Ex, Paris, France

**Keywords:** iron, hepcidin, mouse model, ferritin, lung, inflammation, alveolar macrophages, ferroportin

## Abstract

Pulmonary iron excess is deleterious and contributes to a range of chronic and acute inflammatory diseases. Optimal lung iron concentration is maintained through dynamic regulation of iron transport and storage proteins. The iron-regulatory hormone hepcidin is also expressed in the lung. In order to better understand the interactions between iron-associated molecules and the hepcidin-ferroportin axis in lung iron balance, we examined lung physiology and inflammatory responses in two murine models of systemic iron-loading, either hepcidin knock-out (Hepc KO) or liver-specific hepcidin KO mice (Hepc KOliv), which do (Hepc KOliv) or do not (Hepc KO) express lung hepcidin. We have found that increased plasma iron in Hepc KO mice is associated with increased pulmonary iron levels, consistent with increased cellular iron uptake by pulmonary epithelial cells, together with an increase at the apical membrane of the cells of the iron exporter ferroportin, consistent with increased iron export in the alveoli. Subsequently, alveolar macrophages (AM) accumulate iron in a non-toxic form and this is associated with elevated production of ferritin. The accumulation of iron in the lung macrophages of hepcidin KO mice contrasts with splenic and hepatic macrophages which contain low iron levels as we have previously reported. Hepc KOliv mice with liver-specific hepcidin deficiency demonstrated same pulmonary iron overload profile as the Hepc KO mice, suggesting that pulmonary hepcidin is not critical in maintaining local iron homeostasis. In addition, the high iron load in the lung of Hepc KO mice does not appear to enhance acute lung inflammation or injury. Lastly, we have shown that intraperitoneal LPS injection is not associated with pulmonary hepcidin induction, despite high levels of inflammatory cytokines. However, intranasal LPS injection stimulates a hepcidin response, likely derived from AM, and alters pulmonary iron content in Hepc KO mice.

## Introduction

Although iron is essential for many cellular processes, an excess of iron can be deleterious, as it triggers the production of free radicals (Ray et al., 2012). In mammals, iron homeostasis is complex. It is dependent on both the tightly-regulated absorption of dietary iron by duodenal enterocytes, and recycling of iron from senescent erythrocytes by macrophages, the source of most serum iron.

Disorders of iron homeostasis are among the most common diseases occurring in humans. The imbalance between iron absorption and iron loss underlies conditions ranging from iron deficiency anemia to hereditary hemochromatosis (HH, iron overload diseases) (Gozzelino and Arosio, 2016; Guo et al., 2016). The molecular pathways that achieve iron balance, both at the cellular and systemic levels, have only recently been well-characterized.

At the cellular level, iron homeostasis is orchestrated by the Iron Regulatory Proteins (IRP)1 and 2. This mechanism involves the binding of the IRPs to RNA stem-loop structures, called Iron-Responsive Elements (IREs). This occurs in the untranslated region of target mRNAs encoding proteins involved in iron uptake, including Divalent Metal Transporter 1 (DMT1, *Slc11a2*) and transferrin receptor 1 (TfR1, *Tfrc*); iron storage, such as ferritin (L-ferritin, *Ftl1* and H-ferritin, *Fth1*); and iron export, as ferroportin (*Slc40A1*), thereby controlling mRNA stability or translation (Anderson et al., 2012; Zhang et al., 2014).

Systemic iron homeostasis is regulated by the circulating peptide hormone hepcidin *(encoded by Hamp)*. Hepcidin controls plasma iron concentration by inhibiting cellular iron efflux through binding and induction of the degradation of ferroportin, the only known cellular iron exporter (Ganz and Nemeth, 2012). Hepcidin is produced predominantly in the liver, and its synthesis is tightly regulated. It is induced by plasma and liver iron to maintain stable body iron levels, and suppressed by erythroid activity to ensure adequate iron supply for erythropoiesis (Ganz and Nemeth, 2012). Accordingly, Hepc KO mice (Lesbordes-Brion et al., 2006), as well as Hepc KOliv mice (Zumerle et al., 2014), develop a severe iron overload phenotype with elevated iron levels in the plasma, liver and pancreas, and iron deficiency in macrophages of the spleen and the liver.

While hepcidin is now recognized as the key iron regulatory hormone, it was originally identified as a cationic antimicrobial peptide (AMP), due to its close structural similarity to the beta defensins, and its ability to kill bacteria *in vitro* (Krause et al., 2000; Park et al., 2001; Houamel et al., 2016). Related to its function as an AMP, *Hamp* is induced by infection and inflammation through Toll Like Receptors (TLR) and inflammatory cytokines (Ganz and Nemeth, 2012).

The characterization of hepcidin has provided significant information around the mechanisms of maintenance of systemic iron homeostasis. However, hepcidin is also expressed in tissues not associated with the regulation of systemic iron levels, which has raised the possibility of a role for the hepcidin-ferroportin axis at the individual tissue level. A cell-specific role for hepcidin was recently reported in cardiac iron homeostasis by Lakhal-Littleton et al. who demonstrated that mice with a cardiomyocyte-specific deletion of hepcidin develop fatal contractile and metabolic dysfunction as a consequence of cardiomyocyte iron deficiency (Lakhal-Littleton et al., 2016).

While the majority of proteins important in iron homeostasis are expressed in the lung, including DMT1, ferroportin, ferritin (Ghio, 2009), and hepcidin (Nguyen et al., 2006; Frazier et al., 2011; Sow et al., 2011; Chen Q. X. et al., 2014; Giorgi et al., 2015; Michels et al., 2017), the mechanisms of pulmonary iron handling remain unclear.

In lung tissue, iron homeostasis would seem to be critical, in view of its unique position with regards to antimicrobial defense, as the lung is the principle entry-point for many pathogens, and iron detoxification, as the lung is continuously exposed to high oxygen concentrations and unbound iron particles. In addition, several studies have highlighted profound disruption to pulmonary iron-handling, particularly iron accumulation, in acute and chronic lung diseases (Ghio, 2009).

In this study, we have investigated the mechanisms of intrinsic pulmonary iron handling, and the potential impact of iron dysregulation in lung pathophysiology. We have utilized two distinct mouse models of systemic iron overload, Hepc KO (Lesbordes-Brion et al., 2006) and Hepc KOliv (Hepc KOliv). The latter has a hepatocyte-specific deletion of hepcidin (Zumerle et al., 2014).

## Materials and methods

### Animals

Mice were cared for in accordance with the European convention for the protection of laboratory animals. Animal studies received approval from the Regional Ethics Committee for Animal Experimentation of University Paris Descartes.

Animals were given free access to tap water and a standard laboratory mouse chow diet (AO3, iron content 280 mg/kg, UAR, France). To generate homozygous hepcidin knock-out (Hepc KO) and homozygous wild-type (WT) control mice in the same litters, heterozygous Hepc KO mice were mated and their progeny were used for experiments (Lesbordes-Brion et al., 2006).

Liver-specific hepcidin knock-out (Hepc KOliv) mice as previously described (Zumerle et al., 2014) were used in this study. Both Hepc KO and Hepc KOliv mice are on a C57BL/6 background.

Analysis of the lung phenotype was performed in relatively old mutant male mice (25–35 weeks old) to increase the possibility of detecting any lung iron abnormalities associated to the tardive appearance of iron-loaded alveolar macrophages (AM).

For comparative studies, 40 week-old HFE KO mice (a kind gift from Dr Nancy Andrews) were used (Levy et al., 2000).

At sacrifice, before tissue harvesting, hearts were perfused with 10 mL of PBS.

### Iron and plasma measurements

Plasma and tissue iron content were measured on an Olympus AU400 automat using a photometric colorimetric method. Tissue iron concentrations are presented relative to wet weight. For iron staining, tissues were fixed in 4% formaldehyde and embedded in paraffin. Slides were stained with Perls' Prussian blue and a nuclear fast red counterstain using standard procedures.

### LPS treatment

Inflammation was induced by intraperitoneal (IP) injection of LPS from *E. coli* O111:B4, 2 mg/kg (Deschemin and Vaulont, 2013) and mice were sacrificed 6 h later. Control mice were injected with saline solution. Intranasal instillation was performed on anesthetized mice [ketamine (4 mg/kg) and xylazine (0.4 mg/kg) i.p.] by placing 20 μg of LPS from *E. coli* O111:B4, 2 / 50 μL saline solution in the nostrils (Szarka et al., 1997). Control mice received 50 μL of sterile saline solution. The animals were sacrificed 5 h following intranasal administration.

### Myeloperoxidase activity

Myeloperoxidase (MPO) activity was assessed in lung tissues by the spectrophotometric assay based on MPO-catalyzed oxidation of 3,39,5,59-tetramethylbenzidine (TMB) by H_2_O_2_ (Pulli et al., 2013). Samples were ground with tungsten beads and homogenized in a 0.2% cetyltrimethylammonium bromide (CETAB) buffer, pH 6.0 (Sigma) and then centrifuged at 10,000 g for 15 min at 4°C. The pellets were then resuspended in CETAB buffer, sonicated, and centrifuged. Protein concentration was determined using the Pierce BCA Protein assay kit (Thermo Scientific). Each sample of 90 μg of protein was resuspended in 100 μL of citrate buffer (10 mM, pH 5). For each sample, 30 μL of supernatant was then combined with 45 μL H_2_O_2_ (3 mM), 45 μL TMB solution (3 mM), and 45 μL citrate buffer (10 mM). After 15 min at room temperature, the reaction was stopped by adding 90 μL H_2_SO_4_ (4N) and the absorbance was measured at 450 nm to estimate MPO activity.

### Reverse transcription and real-time PCR

RNA extraction, reverse transcription, and real-time PCR were performed as previously described (Lenoir et al., 2011). Briefly, total RNA was isolated with TriReagent (Sigma), and subsequent cDNA synthesis was performed with the High Capacity cDNA Starter Kit, (Applied Biosystems) according to the manufacturer's instructions. Real-time PCR was performed in a LightCycler 480 Instrument II (Roche) using the SYBR Green PCR mix (Roche) in accordance with the MIQE guidelines (Bustin et al., 2009). Relative mRNA expression levels were determined by the second-derivative maximum method with the LightCycler 480 analysis software. All samples were normalized to the threshold cycle value for cyclophilin-A (*Ppia*). Primer sequences used for this study are provided in Figure S1.

### Western blot (WB)

Membrane and cytosolic fractions were prepared as previously described (Deschemin and Vaulont, 2013). Total lysates from lung, spleen and alveolar macrophages were also prepared. In brief, tissues were homogenized in lysis buffer (50 mM Tris, pH 7.4, 1% Triton X-100, 150 mM NaCl, 10% glycerol, 50 mM NaF, 5 mM sodium pyrophosphate, 1 mM Na_3_VO_4_, 25 mM sodium-β-glycerophosphate, 1 mM DTT, 0.5 mM PMSF), with protease inhibitors, sonicated, and centrifuged at 10,000 rpm for 10 min.

Samples were analyzed by SDS-PAGE and transferred onto a nitrocellulose membrane in Tris/glycine buffer. Blocking of the membrane was performed with 10 mM Tris-buffered saline (pH 7.4), 0.05% Tween 20 (TBST), and 5% (w/v) non-fat milk powder. All primary antibodies were incubated with the membrane overnight, at 4°C, on a rocking platform. Membranes were washed in TBST and probed with the appropriate secondary antibody in TBST + 5% (w/v) non-fat milk powder, for 1 h, at room temperature, on a rocking platform. Membranes were thoroughly washed and chemiluminescence was detected with Supersignal West Pico, Supersignal West Dura substrates (Thermo). Proteins were visualized with Image Quant Las4000 mini (GE Healthcare). The following antibodies were used: anti-DMT1 (gift from FCH, dilution 1/500 (Canonne-Hergaux and Gros, 2002; Canonne-Hergaux et al., 2006); anti-ferroportin (MTP11-A from Alpha Diagnostic; dilution 1/500) (Lakhal-Littleton et al., 2016); anti-L-ferritin (SAB2500431 from Sigma-Aldrich; dilution 1/500), anti-transferrin receptor 1 (13–6800 from Invitrogen, clone H68.4, dilution 1/500) (Barrientos et al., 2015) and anti-β-actin (Ascites fluid A5316 from Sigma-Aldrich, dilution 1/6,000). Secondary antibodies used were anti-rabbit, anti-goat or anti-mouse (Calbiochem, dilution 1/10,000), as appropriate.

Densitometry of the immunoblots was performed using ImageJ software.

### Immunochemistry

Tissues were fixed in 4% formaldehyde, and paraffin-embedded. Immunostaining was performed on 4 μm thick dewaxed tissue sections, boiled in pH6 citrate buffer for 15 min. Endogenous peroxidases were neutralized by 3% H_2_O_2_ treatment for 20 min. Tissues were permeabilized for 20 min (0.5% Triton X-100-PBS) then blocked for 30 min at room temperature (3% BSA, 0.1% Triton X-100, 10% normal goat serum). Primary antibody against ferroportin (MTP11-A from Alpha Diagnostic) was diluted 1:50 before use (3% BSA, 0.1% Triton X-100, 1% normal goat serum) and incubated over night at 4°C. As a negative control, we performed immunohistochemistry without the addition of the primary antibody (Figure S2). We applied HRP-conjugated secondary antibodies with the ImmPACT NovaRED Peroxidase Substrate Kit (Vector Laboratories, Burlingame, CA, USA) according to the manufacturer's recommendations. Counterstaining was performed with Nuclear Fast Red (Vector Laboratories).

### Bronchoalveolar lavage (BAL)

For BAL cell harvest, mice were sacrificed with ketamine (90 mg/kg) and xylazine (10 mg/kg) IP. The trachea was cannulated, and the lungs lavaged with a total of 3 mL of PBS/1.5 mM EDTA in 0.5 mL aliquots. Lavage aliquots for each animal were pooled, and the cell pellet collected by centrifugation. Alveolar macrophages (AM), identified as CD64+ using an ACCURI C6 flow cytometer, were shown to represent more than 95% of the cellular population of the suspension. Perls' Prussian blue staining was performed on the cytospin preparations.

For RNA and protein analysis, the cells were seeded in 24- and 6-well plastic culture dishes, respectively, and the AM purified by adherence at 37°C, for 2 h in a 5% CO_2_ humidified incubator, and any non-adherent cells were removed by washing prior to subsequent extraction. LPS treatments were performed for 6 h with 100 ng/mL LPS.

### Statistical analysis

Statistical analyses, unless otherwise stated, were performed using Student's *t*-test (unpaired, two tailed). *P* < 0.05 were considered statistically significant.

## Results

### Hepc KO mice demonstrate elevated iron loading in the lung

**Figure 1:**
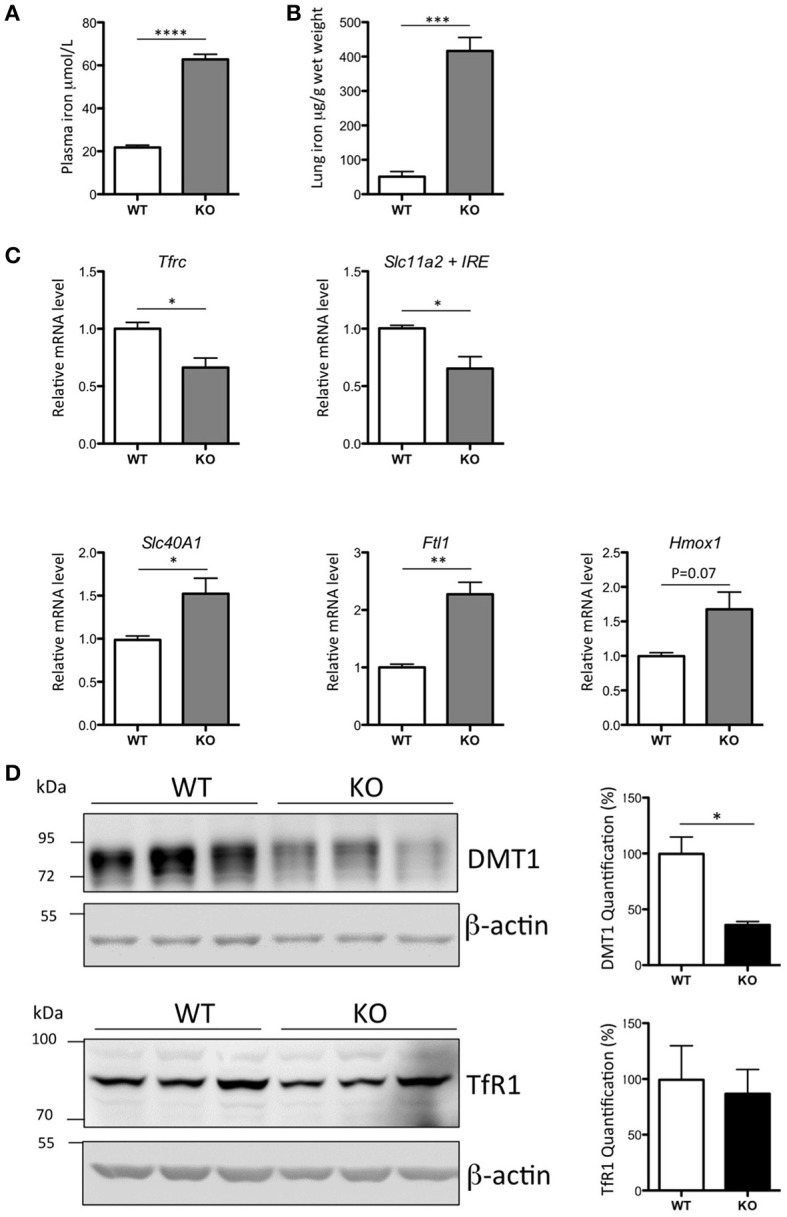
Iron load phenotype in the lung of WT and Hepc KO mice: Plasma iron in μM **(A)** and lung iron in μg/g wet tissue **(B)**. Pulmonary *Tfrc, Slc11a2* + IRE, *Slc40A1, Ftl1*, and *Hmox1* mRNA levels relative to *Ppia* analyzed by real-time PCR. Changes are expressed relative to WT mice **(C)**. Lung DMT1 and TfR1 protein levels analyzed by WB using proteins from membrane enriched-fractions **(D)**. Expression is normalized to beta actin and quantified using Image J. Quantification of the blots are presented in % (100% set for WT). Error bars represent SEM for *n* = 3 mice in each group. Statistical significance is indicated by asterisks (^*^p < 0.05, ^**^p < 0.01, ^***^p < 0.001, ^****^p < 0.0001). Similar results were obtained in at least two independent experiments.

The Hepc KO mice demonstrated a 3-fold increase of plasma iron (Figure 1A), an 8-fold increase of iron in the lung (Figure 1B), and a lung mRNA expression profile characteristic of iron loaded tissue (Figure 1C). As has been previously reported in the liver of Hepc KO mice (Figure S3A), we noted a significant decrease in *Tfrc* and *Slc11a2*, and an increase in *Slc40A1* and *Ftl1* mRNA levels in the lung. There was no significant change in the expression levels of *Hmox1* mRNA.

Of note, while the decrease in *Slc11a2* mRNA level was accompanied by a concomitant decrease in the protein (Figure 1D), there was no relative decrease in the level of TfR1 protein, suggesting an additional mechanism of post-transcriptional regulation of this receptor in the lung.

**Figure 2:**
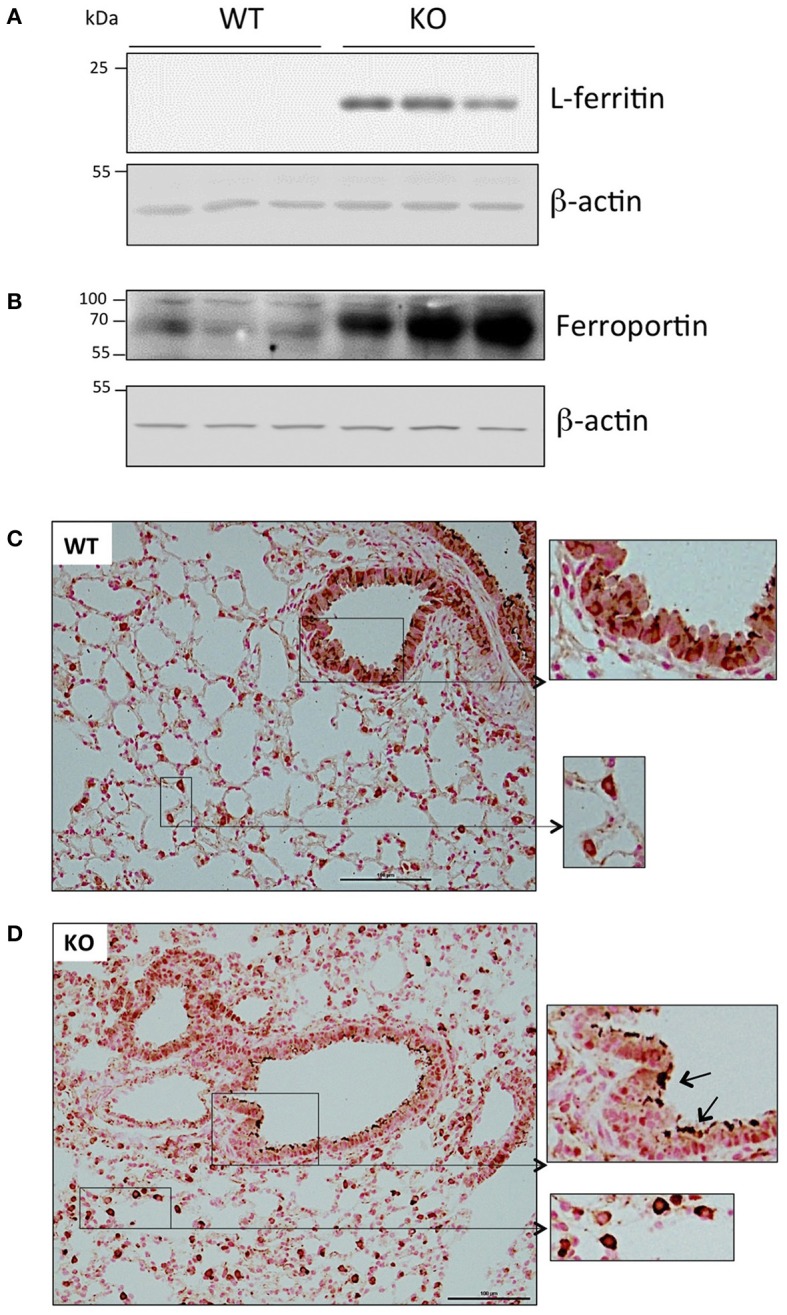
Ferritin and ferroportin expression in the lung of WT and Hepc KO mice: L-ferritin **(A)** and ferroportin **(B)** protein levels analyzed by WB using proteins from cytosolic and membrane enriched-fractions, respectively. Ferroportin immunostaining of lung sections from WT **(C)** and Hepc KO **(D)** mice. The scale bars indicate 100 μm and the insets present a two-fold enlargement. Arrows indicate the strong ferroportin staining detected in the apical membrane of the Hepc KO mice.

At the protein level, we also detected a significant accumulation of the iron storage protein, L-ferritin, in the Hepc KO mice (Figure 2A). This was most likely due to the slight increase of *Ftl1* mRNA expression, and the derepression of *Ftl1* mRNA translation mediated by iron accumulation-induced IRP inactivation (Anderson et al., 2012; Zhang et al., 2014). Ferroportin was detected in membrane preparations of both WT and Hepc KO mice, but it was seen at a much higher level in the KO mice (Figure 2B). This is likely caused by the absence of hepcidin, and, as for L-ferritin, the involvement of the IRE-IRP regulatory system. Similar patterns of L-ferritin and ferroportin accumulation have previously been demonstrated in the liver of Hepc KO mice (Figure S3B).

Ferroportin distribution was further evaluated by immunochemistry in lung sections. As shown in Figure 2C, confirming the results of Yang et al. ferroportin was detected not only in the bronchial epithelium in WT mice, but also in other cells, likely immune cells of the interstitium and alveolar lumen (Yang et al., 2005). Although we did not detect a significant difference in the overall pattern of ferroportin distribution in the lungs of Hepc KO mice, positive staining was much more pronounced in the macrophages, and the protein was largely found in the apical region of the epithelial cells (Figure 2D).

### Iron loading in the lungs of Hepc KO mice is predominantly due to iron accumulation in epithelial cells and macrophages

**Figure 3:**
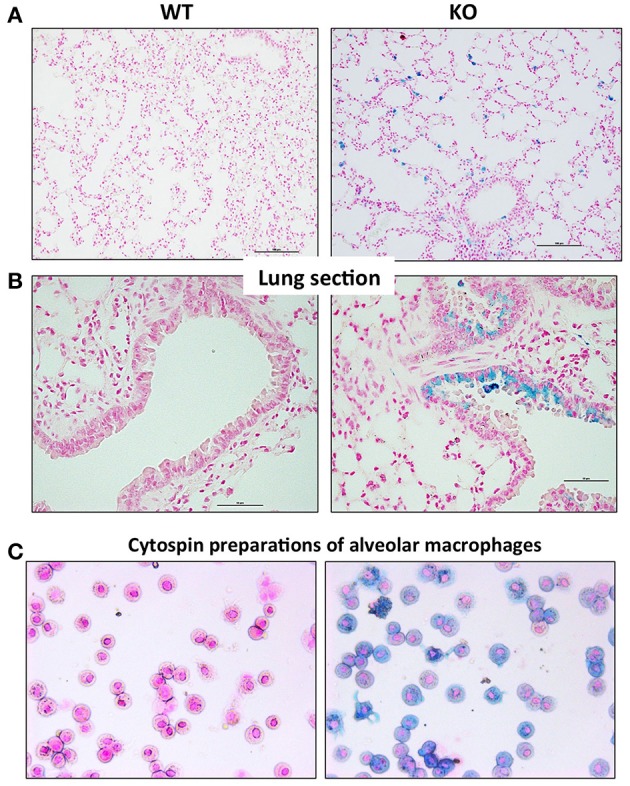
Perls' blue staining of lung section from WT and Hepc KO mice: Iron deposition was visualized using Perls' blue staining in lung section. The scale bars indicate 50 μm **(A)** and 100 μm **(B)**. Representative images of BAL (bronchoalveolar lavages) cytospin slides obtained from WT and Hepc KO mice and stained with Perls' Prussian blue **(C)**.

In order to characterize the pulmonary iron load at the cellular level, lung sections were stained with Perls' Prussian blue. In WT mice, there was no detectable staining present in the tissue. In contrast, very specific staining patterns were observed in the Hepc KO mice, with a large accumulation of iron in alveolar and interstitial immune cells (Figure 3A), and in some bronchial epithelial cells (Figure 3B). It remains unclear what drives iron accumulation in some epithelial cells, while not others. However, the presence of iron in these epithelial cells did not correspond to a reduced amount of ferroportin. On the contrary, the presence of cellular iron was generally found in association with ferroportin (Figure S4), suggesting that iron-induced ferroportin is not sufficient to allow efficient iron export from these cells. Lung iron loading did not demonstrably trigger tissue damage, fibrosis, or inflammation (data not shown).

To further identify the iron-positive immune cells in the alveolus, bronchoalveolar lavages (BAL) were performed. We confirmed by FACS analysis that more than 95% of the cells were AM. The AM were cytospinned and stained with Perls' Prussian blue. As shown in Figure 3C, a large number of the AM isolated from the Hepc KO mice were iron loaded, although at different levels, compared to the WT AM population. This phenotype appears to be unique to this macrophage population, as splenic or hepatic macrophages from Hepc KO mice have been shown to be iron deprived (Lesbordes-Brion et al., 2006). Of note, this phenotype of iron accumulation in the AM develops progressively with age, becoming prominent from 25 weeks old.

### The iron loaded AM of the Hepc KO mice have up-regulated levels of ferritin

**Figure 4:**
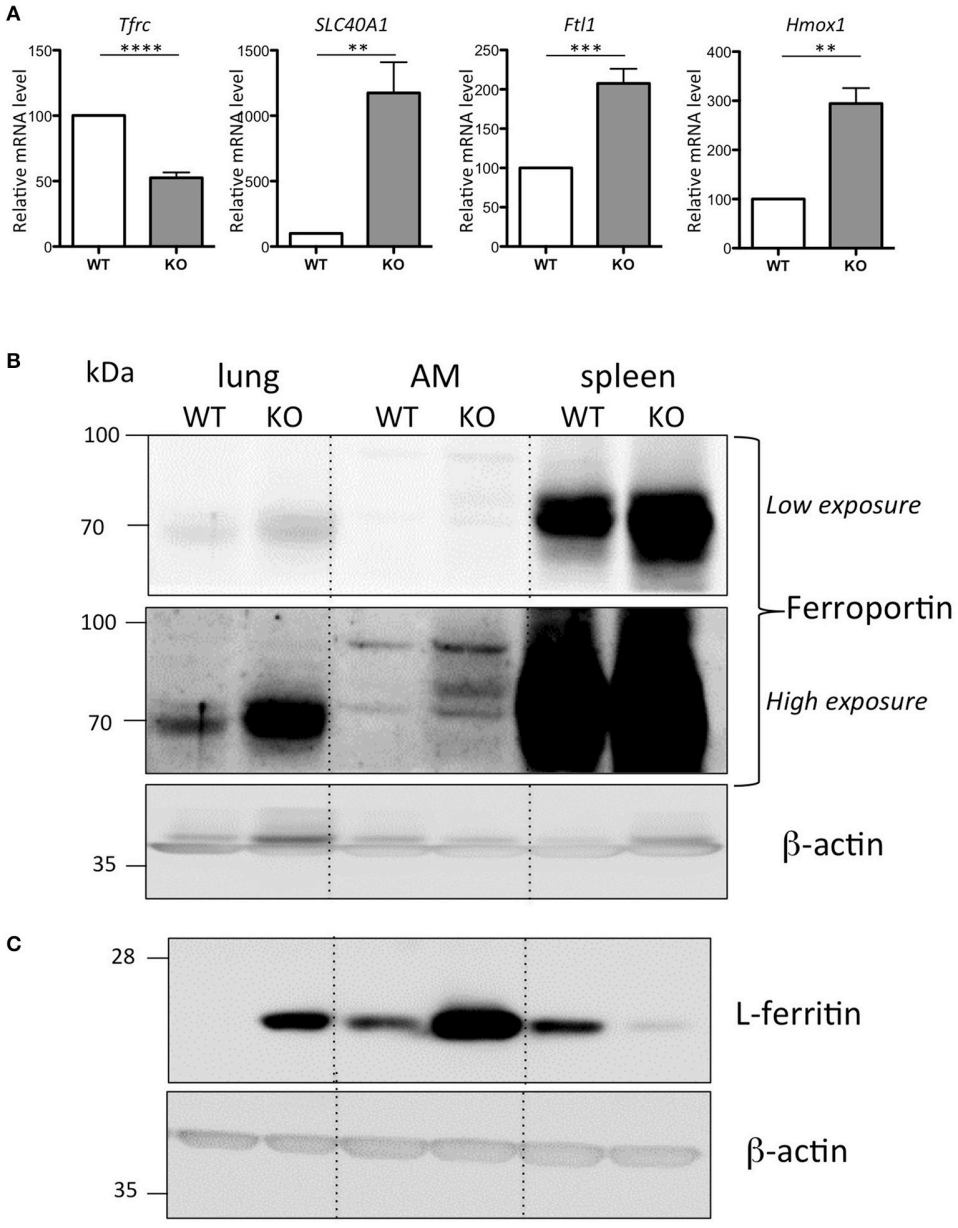
Iron phenotype of the isolated AM from WT and Hepc KO mice: *Tfrc, Slc40A1, Ftl1*, and *Hmox1* mRNA levels in AM relative to *Ppia* analyzed by real-time PCR. The WT AM cells have been used as reference for each single experiment of AM isolation performed on an-age matched couple of WT and Hepc KO mice, and the data are expressed in fold change **(A)**. Error bars represent SEM for *n* = 5 KO mice. Statistical significance is indicated by asterisks (^**^p < 0.01, ^***^p < 0.001, ^****^p < 0.0001). Ferroportin **(B)** and L-ferritin **(C)** protein levels analyzed by WB using total extract proteins from lung, isolated AM and spleen. For AM, each lane corresponds to the pool of extracts from 3 mice per genotype.

Alveolar macrophages (AM) play a central role in defending the lung against pathogens and other environmental challenges, as well as in mediating damage and repair in the lung parenchyma (Hussell and Bell, 2014). We have further characterized the iron-loaded AM isolated from the Hepc KO mice. In response to the increase in iron load accumulation, we observed a down regulation of *Tfrc* and, conversely, an up-regulation of *Slc40A1, Ftl1*, and *Hmox1* mRNA levels in the Hepc KO AM (Figure 4A). In other words, the changes observed in total lung extract induced by iron accumulation in the Hepc KO mice (Figure 1C) were largely reproduced at the level of AM.

Ferroportin accumulation in AM was further confirmed by WB analysis on total cellular lysates. This showed an additional ferroportin band strongly up-regulated in the AM isolated from the Hepc KO mice (high exposure, Figure 4B). Importantly, we found that, consistent with the higher content of iron in the Hepc KO AM compared to WT AM, ferritin levels were higher (Figure 4C). This is in contrast to control lysates from Hepc KO spleen extracts, which demonstrated a down-regulation of L-ferritin, highlighting the iron deficient status of spleen macrophages of these mice (Figure 4C), together with an increase of ferroportin (see low exposure, Figure 4B), as has been previously reported (Lesbordes-Brion et al., 2006; Deschemin and Vaulont, 2013).

In contrast to the iron loaded AM of the Hepc KO mice, Benesova et al. reported iron deficiency in the AM of HFE KO mice (Benesova et al., 2012). This is a mouse model of one of the most common forms of HH, characterized by low hepcidin levels (Pietrangelo, 2010). Interestingly, we did not see the same changes in mRNA levels in Hepc KO AM as in isolated HFE AM (Figure S5). This strongly suggests that iron, rather than hepcidin deficiency, is the primarily signal responsible for triggering changes in mRNA expression levels observed in the Hepc KO AM.

Together, our results are consistent with the following scenario: elevated plasma iron in the Hepc KO mice leads to increased iron uptake by pulmonary epithelial cells. The iron loaded pulmonary epithelium then up-regulates ferroportin, resulting in increased iron export at the apical membrane and subsequent iron uptake by AM. Iron in the AM is sequestered in a non-toxic form by ferritin, indicating a role for AM in the protection of nearby cells from iron toxicity.

### Iron loading in the lung of Hepc KO mice is predominantly due to liver hepcidin deficiency (and not lung hepcidin)

**Figure 5:**
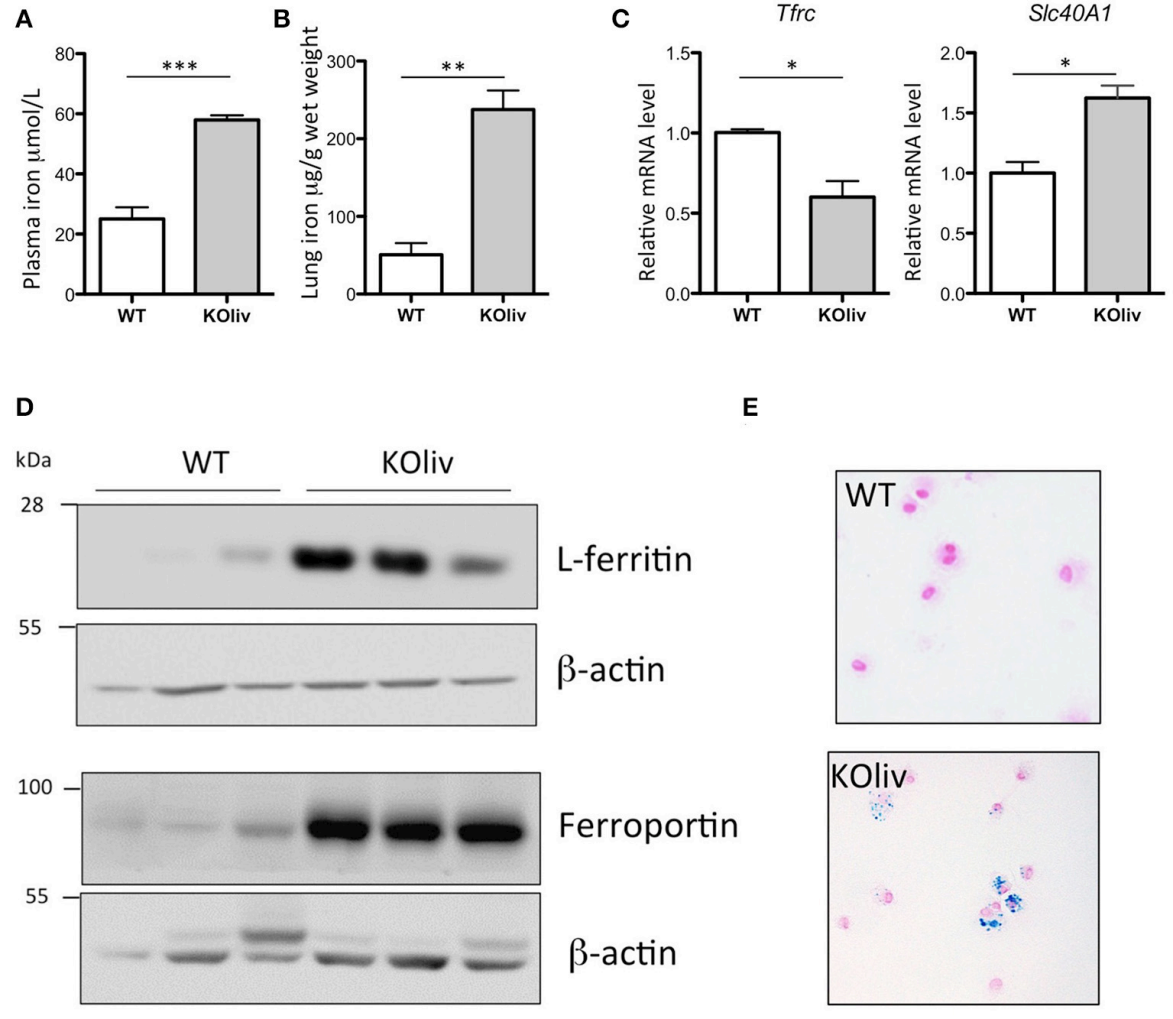
Iron load phenotype in the lung of WT and Hepc KOliv mice: Plasma iron in μM **(A)** and lung iron in μg/g wet tissue **(B)**. Pulmonary *Tfrc*, and *Slc40A1* mRNA levels relative to *Ppia* analyzed by real-time PCR. Changes are expressed relative to WT mice **(C)**. L-ferritin and ferroportin **(D)** protein levels analyzed by WB using proteins from cytosolic and membrane enriched-fractions, respectively. Representative images of BAL (bronchoalveolar lavages) cytospin slides obtained from WT and Hepc KOliv mice and stained with Perls' Prussian blue **(E)**.The set of results obtained with WT and Hepc KOliv mice were reproduced using another serie of animals. Error bars represent SEM for *n* = 3–4 mice in each group. Statistical significance is indicated by asterisks (^*^p < 0.05, ^**^p < 0.01, ^***^p < 0.001).

While hepcidin is expressed predominantly in the liver (Zumerle et al., 2014), hepcidin mRNA can also be found, although at much lower levels, in a number of other tissues, including the lung. We thus evaluated whether pulmonary hepcidin deficiency was involved in the accumulation of iron in the lung of Hepc KO mice by a para- or autocrine mechanism. To answer this question, we have utilized the recently described liver-specific KO mouse model (Hepc KOliv), which resembles the total KO mouse in terms of iron overload phenotype, but which expresses lung hepcidin (Zumerle et al., 2014). The same features of pulmonary iron loading as those described in the total KO mice were observed in these mice, although to a lesser degree. The Hepc KOliv mouse model demonstrated a 2.4-fold increase of plasma iron (Figure 5A), iron accumulation in the lung (Figure 5B), decreased *Tfrc* and increased *Slc40A1* mRNA levels (Figure 5C), an increase of L-ferritin and ferroportin protein levels (Figure 5D), and iron accumulation in the AM (Figure 5E). These results suggest that lung-specific hepcidin is not the primary cause of iron dysregulation in the Hepc KO mice, as these changes were observed in both the presence (Hepc KOliv mice) and absence (Hepc KO mice) of lung-derived hepcidin.

### Inflammatory responses in systemic endotoxin-induced lung injuries are independent of hepcidin

**Figure 6:**
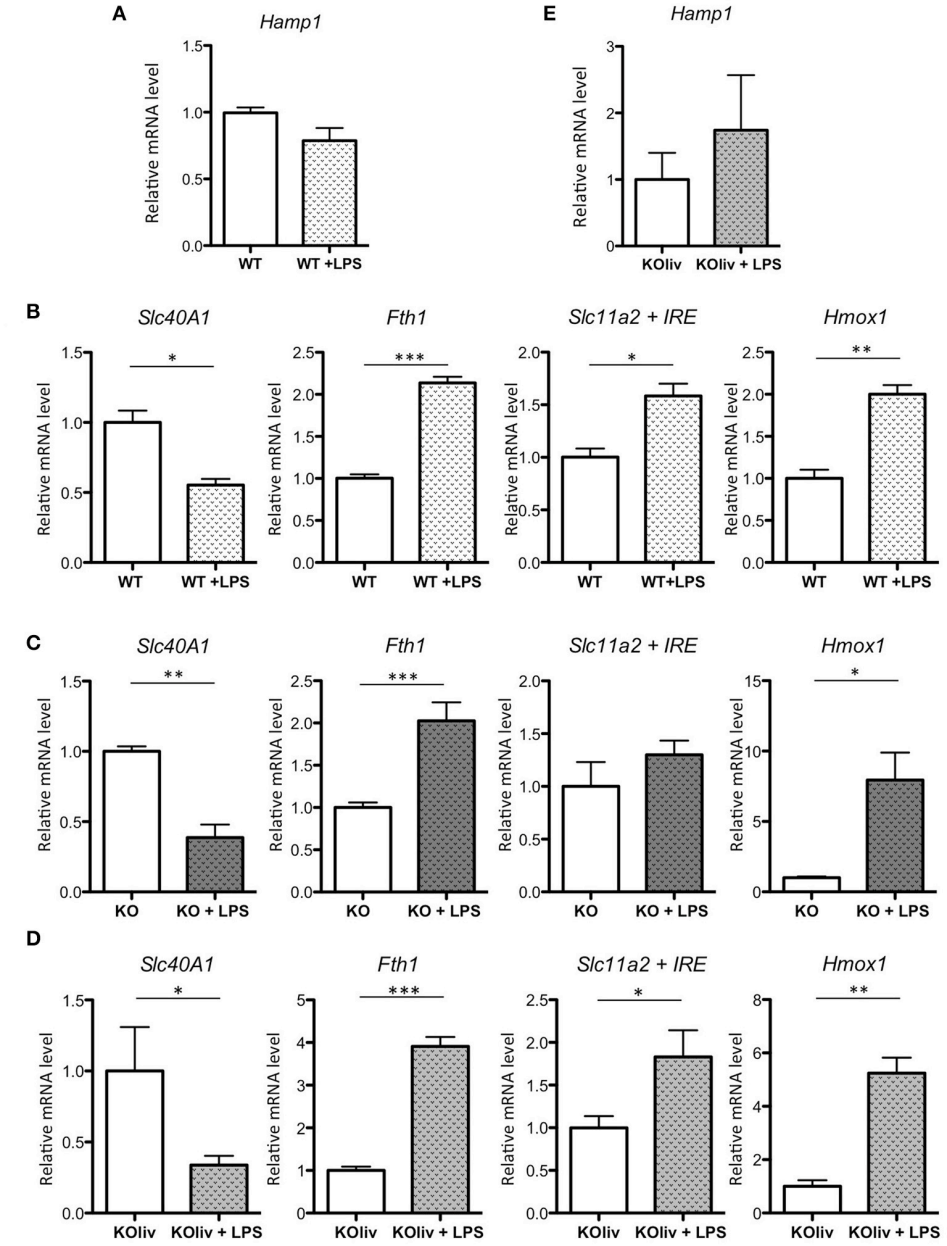
LPS responses in the lung of WT, Hepc KO and Hepc KOliv mice: *Hamp* **(A)**, as well as *Slc40A1, Fth1, Slc11a2* + IRE, and *Hmox1* mRNA levels relative to *Ppia*, analyzed by real-time PCR in the lung of WT mice **(B)**, Hepc KO mice **(C)** or Hepc KOliv **(D)** mice IP injected for 6 h by LPS. Changes are expressed relative to untreated WT or KO mice, respectively. *Hamp* **(E)**, relative to *Ppia*, in the lung of Hepc KOliv mice IP injected for 6 h by LPS. This set of results were reproduced using another serie of WT and Hepc KO mice. Error bars represent SEM for *n* = 3–4 mice in each group. Statistical significance is indicated by asterisks (^*^p < 0.05, ^**^p < 0.01, ^***^p < 0.001).

Acute lung injury is a clinical syndrome characterized by an excessive inflammatory response, carrying severe consequences for tissue integrity and function, and it is associated with high mortality. To determine the role of hepcidin in the pulmonary inflammatory response, we administered gram-negative bacterial endotoxin lipopolysaccharide (LPS) via IP injection to WT and Hepc KO mice. This is a recognized model of acute inflammation causing a transient systemic lung inflammation (Rojas et al., 2005). Following LPS injection of the WT mice, there was a considerable pulmonary inflammatory response (with the classical increase in inflammatory cytokines such as IL6, CXCL1 (also known as keratinocyte-derived chemokine KC), and TNF-alpha (data not shown). However, in contrast to the up-regulation of hepcidin gene expression observed in the liver (Deschemin and Vaulont, 2013), and in other tissues, such as the kidney and the spleen (data not shown), pulmonary *Hamp* gene expression was not induced (Figure 6A). With respect to the other iron-related genes, we observed a similar response in the lung to that seen in the liver (Deschemin and Vaulont, 2013), specifically a decrease of *Slc40A1*, and an increase of *Fth1, Slc11a2*, and *Hmox1* mRNA levels, after LPS injection (Figure 6B). Interestingly, these responses were also observed in the lung of Hepc KO mice (Figure 6C) and Hepc KOliv mice (Figure 6D). The Hepc KOliv mice also demonstrated an absence of *Hamp* up-regulation in the lung in response to IP LPS (Figure 6E). Overall, these results suggest that pulmonary and liver hepcidin are not necessary for the regulation of iron-related genes during inflammation, and that iron loading in the lung is not globally impacting the inflammatory response.

Neutrophil recruitment at the site of inflammation is a hallmark of the acute phase of lung injury (Grommes and Soehnlein, 2011). As macrophage and systemic iron may affect neutrophil migration (Kartikasari et al., 2009; Wang et al., 2009; Benesova et al., 2012), we measured lung neutrophil MPO. This is the most abundant proinflammatory enzyme stored in the granules of neutrophils (Pulli et al., 2013), and is an effective surrogate marker for the quantification of tissue neutrophils. MPO activity was largely increased after LPS injection (Figure S6A), although the level was similar to that seen in the lungs of both WT and KO LPS-treated mice. This suggests that the lung iron disturbance in the Hepc KO mice does not significantly impact LPS-mediated neutrophil recruitment, at least during the first stages of activation and sequestration of neutrophils from the blood to the interstitium. We also investigated expression of the two most important chemokines involved in neutrophil recruitment to the lung in rodents (Grommes and Soehnlein, 2011), CXCL1 and CXCL2 (also known as macrophage inflammatory protein-2, MIP-2). Again, we found similar mRNA expression levels of these chemokines in the lung tissue of both WT and KO LPS-challenged mice (Figure S6B).

### Direct LPS airway exposure induces a decrease in pulmonary iron levels in Hepc KO mice

**Figure 7:**
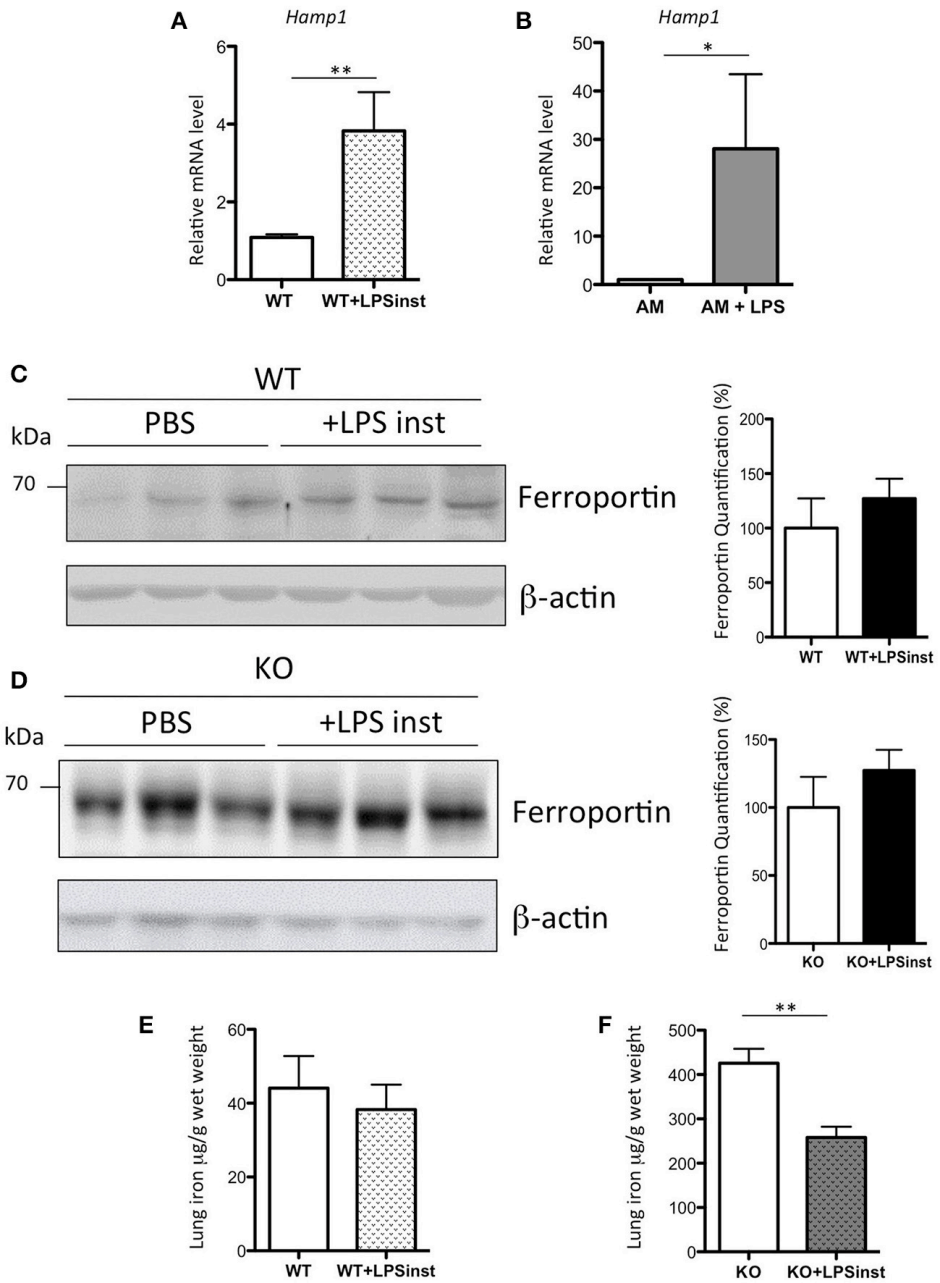
Hepcidin, ferroportin, and iron responses after intranasal LPS instillation in WT and Hepc KO mice: *Hamp*, relative to *Ppia*, analyzed by real-time PCR, in the lung of mice given intranasal LPS infusion for 5 h **(A)** and in purified AM treated for 6 h with LPS **(B)**. Changes are expressed relative to PBS treated mice or untreated AM, respectively. Error bars represent SEM for *n* = 3–5 mice in each group. Statistical significance is indicated by asterisks (^*^p < 0.05, ^**^p < 0.01). Ferroportin protein levels analyzed by WB using proteins from membrane enriched-fractions isolated from the lung of WT **(C)** or Hepc KO mice **(D)** given intranasal LPS infusion for 5 h. Quantifications of the blots are presented in % (100% set for WT and KO mice infused with PBS, respectively). Lung iron content in the lung of WT **(E)** or Hepc KO mice **(F)** given intranasal LPS infusion for 5 h. Statistical significance is indicated by asterisks (^**^p < 0.01).

Systemic administration of sub-lethal dose of LPS results in the release of a cytokine flood into the circulation (Rojas et al., 2005), and in particular IL6, which is a potent inducer of *Hamp* expression in the liver (Ganz and Nemeth, 2015). Thus, the absence of an increase in *Hamp* expression in the lung was rather surprising. Additionally, Benesova et al. have previously reported a 2.5-fold increase in pulmonary *Hamp* expression upon intratracheal LPS injection (Benesova et al., 2012). As the route and the dose of LPS injection are critical to the activation of distinct subsets of cells, we had anticipated that the response of hepcidin to airway LPS injection to be the result of AM activation (and possibly other immune cells), rather than epithelial cells. To test this hypothesis, LPS was delivered to the lung tissue of WT mice through intranasal infusion, as described previously (Szarka et al., 1997). In contrast to the administration of IP LPS, a significant 4-fold increase of lung *Hamp* expression was observed 5 h after LPS infusion, as shown in Figure 7A. The effect of LPS was further tested *in vitro*. In each of the epithelial cell lines tested, we found very low to undetectable levels of *Hamp* expression, and we were unable to induce up-regulation of *Hamp* gene expression by LPS treatment of these cells (data not shown). In contrast, however, we observed that LPS added to isolated AM could induce a 28-fold increase of *Hamp* gene expression (Figure 7B).

To further understand the effect of intranasal LPS-induced hepcidin expression on iron homeostasis, we investigated the level of ferroportin expression in the lung tissue of LPS-treated animals. We found that there was no change in ferroportin protein levels (Figure 7C), despite the large increase in *Hamp* gene expression. Correspondingly, levels of iron in the lung were similar in LPS-treated animals compared to controls (Figure 7E). To further evaluate the role of hepcidin in pulmonary iron homeostasis, Hepc KO mice were infused with LPS under the same conditions. While ferroportin levels were found to be similar in PBS and LPS infused KO mice (Figure 7D), LPS-infused KO animals demonstrated a striking and significant decrease in pulmonary iron levels (Figure 7F), suggesting regulation of lung-specific iron by a hepcidin-ferroportin independent mechanism.

## Discussion

In this study, we have characterized the pulmonary iron-related phenotype of Hepc KO mice. We have shown that Hepc KO mice present with an accumulation of iron in the lung, and we propose that this is a result of an increase in uptake of circulating iron by epithelial cells. We have further identified an accumulation of iron in the alveoli, particularly in the AM, in conjunction with elevated ferroportin levels at the apical membrane of the epithelial cells (Figure S7).

As with any cell type, net iron accumulation in epithelial cells and AM may be the result of either increased iron uptake and/or decreased iron export. In this instance we favor the first hypothesis, as we have found, in both cell types, an increase in expression of the iron exporter ferroportin, which is likely associated with elevated iron deposition (Figure 2). In conditions of iron overload, high levels of plasma iron are associated with the presence of non-transferrin bound iron (NTBI), which can be efficiently taken up by tissues (Brissot et al., 2012). DMT1 and the metal-ion transporters of the ZIP family (first characterized by their ability to transport zinc) have been indicated in this NTBI transport mechanism (Wang et al., 2002; Bresgen and Eckl, 2015; Giorgi et al., 2015; Jenkitkasemwong et al., 2015). However, we found similar pulmonary mRNA levels between Hepc KO and WT mice for *Slc11a2*-IRE isoform (the isoform proposed by Wang et al. to be responsible for elevated iron transport in the lung of rats treated with ferric ammonium citrate (Wang et al., 2002), and a decrease in DMT1 protein levels (Figure 1D). Additionally, we did not observe any changes in the mRNA expression levels of ZIP 8, which is typically abundant in the lung (Wang et al., 2012), or ZIP 14, which is involved in NTBI uptake by liver and pancreas (Jenkitkasemwong et al., 2015) (data not shown). In fact, the sustained presence of TfR1 protein at the membrane, despite decreased *Tfrc* mRNA levels (Figures 1C,D), could contribute to the increase of iron content in the lung of the Hepc KO mice.

One particularly interesting finding from this study is that AM behave very differently with regards to iron handling, compared to splenic macrophages, which are typically exposed to high iron flux, as the professional macrophages for the clearance of aged or stressed erythrocytes. Ferroportin stabilization in the macrophages of the spleen (and liver) of the Hepc KO mice triggers increased iron export, leading to iron deficiency in macrophages of these tissues (Lesbordes-Brion et al., 2006; Deschemin and Vaulont, 2013). In contrast, however, we have reported here that over-expression of ferroportin in the AM was associated with iron accumulation in these cells. Of note, the ferroportin species detected in the AM was of a higher molecular mass than that seen in the whole lung, indicating the possibility of post-translational modification to this protein, as has been previously reported (Canonne-Hergaux et al., 2006). This could consequently affect both the subcellular localization and the function of the protein.

Although somewhat intensified by the systemic iron overload, pulmonary iron handling in Hepc KO mice is similar to that observed in WT mice. Indeed, the epithelium of the lung has not evolved to provide iron to the body, unlike the iron-absorbing intestinal epithelium, but rather to control iron-catalyzed oxidative stress and to preserve lung function. In conjunction with this function of iron detoxification, iron is taken up by pulmonary cells and further passed to macrophages of the alveoli, where it is safely stored (Yang et al., 2002; Ghio, 2009). This routing of iron from the epithelia to the AM in experimentally iron-loaded mice has been recently reported by Giorgi et al. (2015), and an increase of AM iron content due to increased iron supplementation in the diet of rats was also reported (Ward et al., 2009).

While mechanisms of iron-handling have been described as altered in several acute and chronic diseases (Ghio, 2009), there are few reports of iron-induced lung disturbance in iron overload pathologies. In the HFE KO mouse model, which is representative of the most common form of HH, Benesova et al. reported not only that the lung was not iron loaded, but also iron depletion in the AM (Benesova et al., 2012). This phenotype is, according to the authors, explained by low liver and/or pulmonary *Hamp* expression-induced ferroportin stabilization and iron export from this tissue. However, levels of ferroportin were not reported in this study. The difference between the iron loading phenotype in the two mouse models of HH (HFE *vs*. Hepc KO mice) is notable, but it may be explained by the greater increase of plasma iron in the Hepc KO compared to the HFE KO mice (1.4-fold increase in HFE vs. 3-fold increase in Hepc KO mice, as compared to that of matched WT controls), and the persistent, although attenuated, expression of hepatic liver hepcidin expression in the HFE KO mice. Additionally, experiments in the HFE KO mice were conducted in younger mice than those used for our study. Finally, a role of HFE in pulmonary iron balance cannot be ruled out.

Of particular interest, Neves et al. (2017) have recently investigated a different model of HH, generated by introducing a point mutation in the ferroportin exporter that confers resistance to hepcidin (Slc40a1^C326S^ mice) (Altamura et al., 2014). This model closely resembles that of the Hepc KO mice, with increased systemic iron levels, iron depletion in macrophages, and iron deposition in the liver and pancreas. However, while both models presented with pancreatitis caused by iron-loaded acinar cells (Altamura et al., 2014; Lunova et al., 2017), premature death due to severe exocrine pancreatic failure was seen only in the Slc40a1^C326S^ mice. Neves et al. have also reported features of pulmonary iron overload in the Slc40a1^C326S^ mice that are very similar to those we describe here for Hepc KO mice, particularly with respect to our observation of iron-loaded AM and epithelial cells. However, while we were unable to detect an alteration to pulmonary function in the Hepc KO mice (for example, we did not find any differences in blood oxygen saturation between Hepc KO and WT mice, data not shown), Neves et al. reported a distinctive phenotype of restrictive lung disease in the Slc40a1^C326S^ mice, in association with decreased total lung capacity and reduced blood oxygen saturation (Neves et al., 2017). The extent to which this pulmonary phenotype is related to pulmonary iron overload or to the pathophysiological consequences of iron overload in other tissues is unclear. It is well-recognized that severe pancreatitis is associated with multiple organ dysfunction, particularly pulmonary complications (Browne and Pitchumoni, 2006), which may largely contribute to the premature death of the Slc40a1^C326S^ mice. These differences in lung phenotype, in conjunction with the difference in life-span between the animals, highlights that the two mouse models are not phenotypically identical, and strongly suggests additional, distinctive roles for both hepcidin and/or ferroportin.

While there is little doubt that hepcidin is expressed in the lung, it has been difficult to identify the characteristics of the cells expressing the peptide, and its exact mode of regulation. *Hamp* expression has been reported by real-time PCR in lung tissue from mice (Benesova et al., 2012; Li C. et al., 2013; Chen Q. X. et al., 2014) and rats (Li Y. Q. et al., 2013), in human normal pulmonary tissue (Chen Q. et al., 2014), in primary differentiated human bronchial epithelial cells from normal donors (Frazier et al., 2011), and in various bronchial epithelial and lung carcinoma immortalized cell lines (Sow et al., 2011; Chen Q. et al., 2014). Immunohistochemical detection of hepcidin has been reported in mouse lung, in both the epithelium and AM (Chen Q. X. et al., 2014; Giorgi et al., 2015), and in normal human lung (Chen Q. et al., 2014). In this latter study, the authors reported strong positive immunoreactivity to hepcidin in lung macrophages, but almost no immunoreactivity in epithelial cells. However, we were unable, in this study, to demonstrate positive immu nohistochemical staining of hepcidin in the lung tissue of WT mice, using Hepc KO mice as controls.

In the cell lines we tested, *Hamp* expression was very low (H292, H1299, A549), or even undetectable (H460, H358). Interestingly, the human A549 alveolar type II cell line gave the strongest *Hamp* signal, raising the possibility of a role for the alveoli in the production of this peptide. Sow et al. have reported that *Hamp* expression can be increased in A549 alveolar cells by interferon gamma (IFNγ). They suggest that during natural infection, lymphocyte-derived IFNγ may participate in the host defense strategy by up-regulating *Hamp*, which acts as an AMP to reduce iron in the alveolar space (Sow et al., 2011). IFNγ was also shown by Frazier et al. to increase *Hamp* gene expression in differentiated human airway cells (Frazier et al., 2011). Notably, in both of these cell lines, there was a negligible *Hamp* response to LPS stimulation (Frazier et al., 2011; Sow et al., 2011), in agreement with our results. A potential role for of hepcidin as an AMP peptide in pulmonary immune defense mechanisms was recently proposed by Chen Q. X. et al. (2014). In this study, the authors reported that the knockdown of airway epithelial cell-derived hepcidin exacerbated polymicrobial sepsis-induced lung injury and pulmonary bacterial infection (Chen Q. X. et al., 2014). In accord with our results from the Hepc KO mice, Chen et al. demonstrated that the knockdown of hepcidin leads to ferroportin accumulation in both epithelial cells and AM. However, they also reported low intracellular iron content in these AM, as assessed by Perls' blue staining, likely reflective of the short-time effect of ferroportin stabilization, which is distinct from our model of long term ferroportin stabilization.

In contrast to the absence of *Hamp* induction in LPS-treated epithelial cells *in vitro*, we have shown a dramatic increase of *Hamp* expression in LPS-treated AM (Figure 7B), as has been previously reported (Nguyen et al., 2006). While we had expected that the up-regulation of *Hamp* would lead to an increase of iron accumulation, we have demonstrated that induction of *Hamp* has no impact on lung iron load in WT mice infused with LPS. This result may be explained by kinetics (with longer exposure times potentially necessary to produce an observable effect), or by the inability of airway-specific hepcidin to regulate ferroportin levels, as observed in Figure 7C. In keeping with this hypothesis, Frazier et al. reported that IFN-γ-induced hepcidin expression in the airway did not induce changes to cell surface expression of ferroportin (Frazier et al., 2011). Interestingly, following LPS infusion in the Hepc KO mice, we have observed a drastic reduction in pulmonary iron content, compared with PBS infused Hepc KO mice, but without a concomitant change in ferroportin levels. Ferritinophagy is unlikely to be involved in the process of iron reduction in this model, as we have not seen changes in the ferritin levels of the cytosols of PBS or LPS infused Hepc KO mice (data not shown). In addition, the mRNA levels of the iron chaperone Poly(C) Binding Protein Pcbp2, which transports iron to ferroportin (Yanatori et al., 2016), and ceruloplasmin, a ferroxidase enzyme that facilitates iron release from cells (Musci et al., 2014), were found to be similar between the two groups (data not shown). The putative hepcidin-independent mechanisms responsible for this iron reduction are currently being investigated.

In conclusion, our results indicate that in the two models of hepcidin deficiency presented here, an elevated systemic iron load leads to iron accumulation in the lung, in both the epithelium and AM, and that pulmonary hepcidin is not critical to the fundamental mechanism of iron regulation. This study provides further evidence of the resistance to tissue damage and dysfunction during pulmonary iron-overload in hepcidin deficient mouse models, and that pulmonary hepcidin is not required in the inflammatory response of iron-related genes to acute LPS-induced inflammation.

## Author contributions

SV conceived the study and wrote the manuscript. JD, SZ, JM performed experiments. JD, SZ, JM, CP, and SV analyzed and interpreted the data. All authors critically revised the manuscript and approved its final version.

### Conflict of interest statement

The authors declare that the research was conducted in the absence of any commercial or financial relationships that could be construed as a potential conflict of interest.
