# Supplementary material for: Pulmonary Iron Homeostasis in Hepcidin Knockout Mice
Source: Front Physiol. 2017 Oct 17;8:804. doi: 10.3389/fphys.2017.00804 (PMC5650979; doi:10.3389/fphys.2017.00804)
Supplement: Supplementary file 1 [file DataSheet1.pdf]

Supplemental Material

Pulmonary iron homeostasis in hepcidin knockout mice

Jean-Christophe Deschemin[1,2,3,4], Jacques RR Mathieu[1,2,3,4,$], Sara Zumerle[1,2,3,4,$], Carole Peyssonnaux[1,2,3,4] and Sophie Vaulont[1,2,3,4*]

[$] These authors contributed equally to this work

[1] INSERM, U1016, Institut Cochin, Paris, France
[2] CNRS, UMR8104, Paris, France
[3] Université Paris Descartes, Sorbonne Paris Cité, Paris, France
[4] Laboratory of Excellence GR-Ex

* **Correspondence**:
Corresponding Author
sophie.vaulont@inserm.fr

Supplementary Figures

Figure S1

Mouse primers sequences used for real-time PCR

HGNC Symbol	Primer	Sequence (5'–3')
Hamp1 (Hepcidin1)	Forward	CCT ATC TCC ATC AAC AGA T
	Reverse	TGC AAC AGA TAC CAC ACT G
Slc40A1 (Ferroportin)	Forward	TTG CAG GAG TCA TTG CTG CTA
	Reverse	TGG AGT TCT GCA CAC CAT TGA T
Hmox1 (Heme Oxygenase1)	Forward	GTC AAG CAC AGG GTG ACA GA
	Reverse	ATC ACC TGC AGC TCC TCA AA
Ftl (L-Ferritin)	Forward	GGG CCT CCT ACA CCT ACC TC
	Reverse	CTC CTG GGT TTT ACC CCA TT
Fth (H-Ferritin)	Forward	GAC CGA GAT GAT GTG GCT CT
	Reverse	GTG CAC ACT CCA TTG CAT TC
TfrC (Tfr1)	Forward	TCC GCT CGT GGA GAC TAC TT
	Reverse	TCA AGT TCT CCA CTA AAG C
Slc11a2 (DMT1+IRE)	Forward	TGT TTG ATT GCA TTG GGT CTG
	Reverse	CGC TCA GCA GGA CTT TCG AG
Ppia (Cyclophilin A)	Forward	ATG GCA CTG GCG GCA GGT CC
	Reverse	TTG CCA TTC CTG GAC CCA AA
CXCL1	Forward	CAA TGA GCT GCG CTG TCA GTG
	Reverse	CTT GGG GAC ACC TTT TAG CAT C
CXCL2	Forward	CCA AGG GTT GAC TTC AAG AAC
	Reverse	AGC GAG GCA CAT CAG GTA CG

Figure S2

A

B

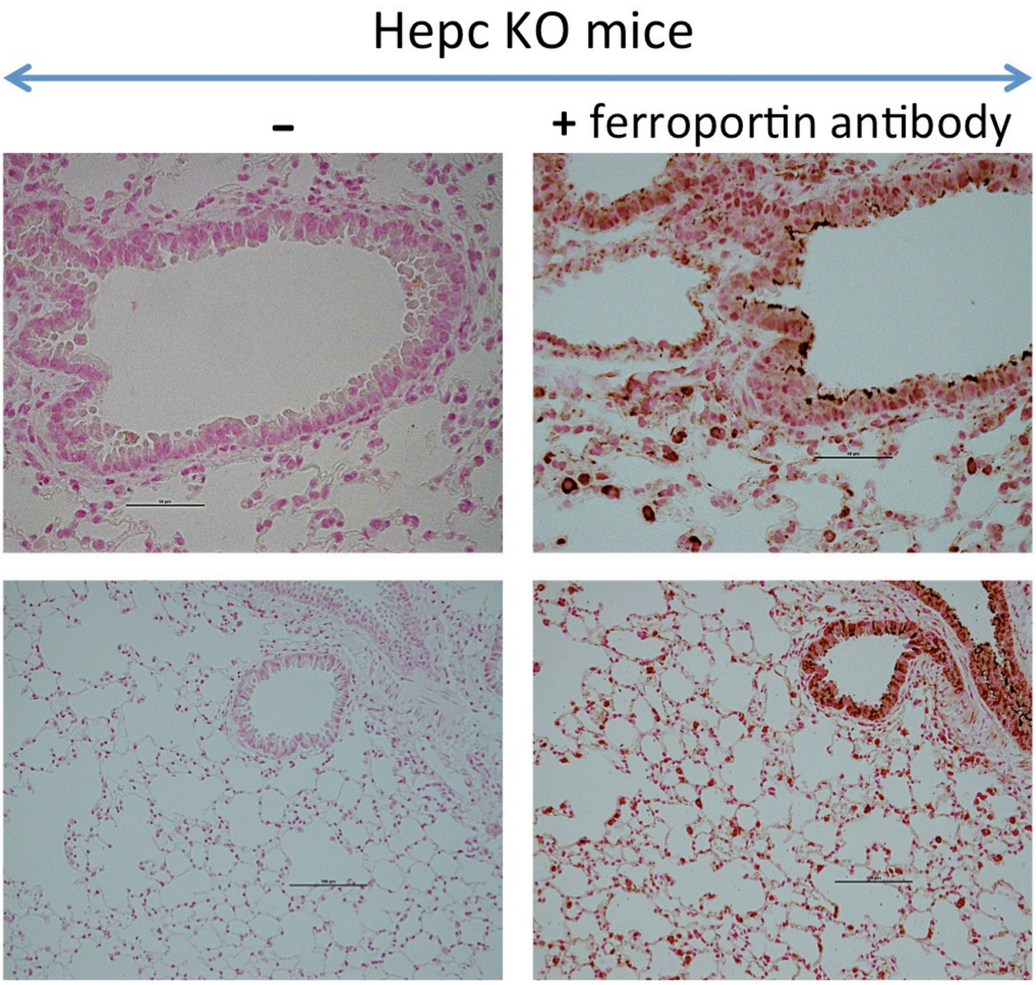

Supplementary Figure 2: Lung section from Hepc KO mice
Immunostaining without (-) or with (+) pimary ferroportin antibody was performed on lung sections from Hepc KO mice. The scale bars indicate 50 μm (A) and 100 μM (B).

Figure S3

A

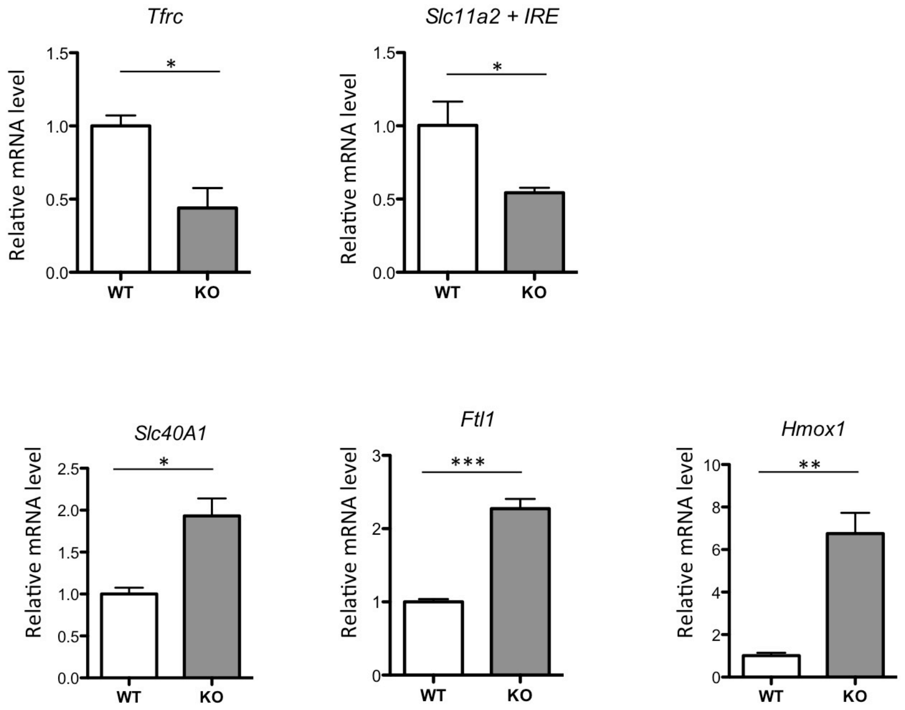

B

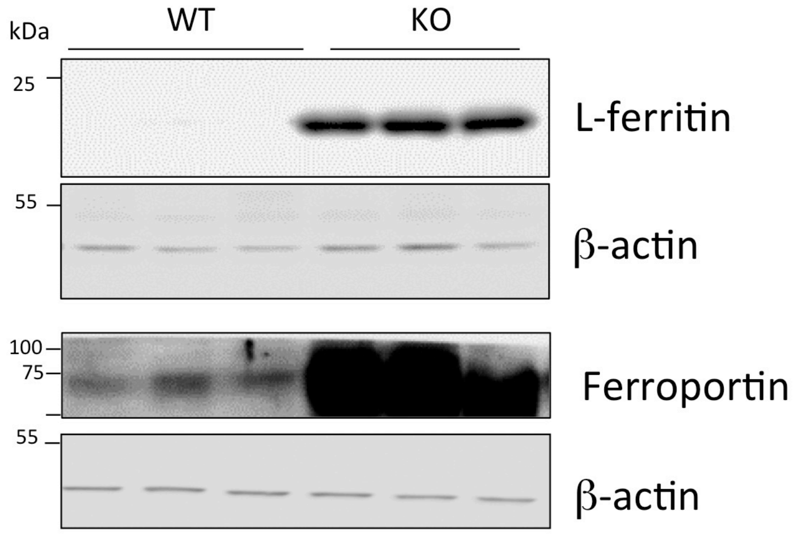

Supplementary Figure 3: Iron load phenotype in the liver of WT and Hepc KO mice:
Hepatic *Tfrc*, *Slc11a2* + IRE, *Slc40A1*, *Ftl1*and *Hmox1*mRNA levels relative to *Ppia* analyzed by real-time PCR. Changes are expressed relative to WT mice (A).
L-ferritin and ferroportin protein levels analyzed by WB using proteins from cytosolic and membrane enriched-fractions, respectively (B).
Error bars represent SEM for n=3 mice in each group. Statistical significance is indicated by asterisks (*p< 0.05, **p< 0.01, ***p< 0.001).

Figure S4

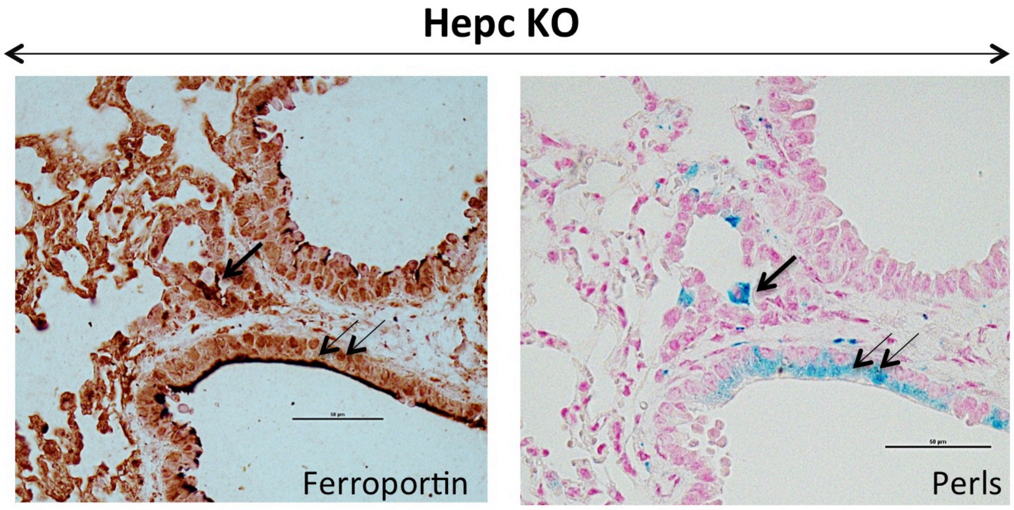

Supplementary Figure 4: Lung staining analysis in WT and Hepc KO mice
Ferroportin immunostaining and Perls' blue staining of lung section from KO mice. Arrows indicate the co-detection of ferroportin and iron in AM and epithelial cells on two consecutives slides. The scale bars indicate 50 µm.

Figure S5

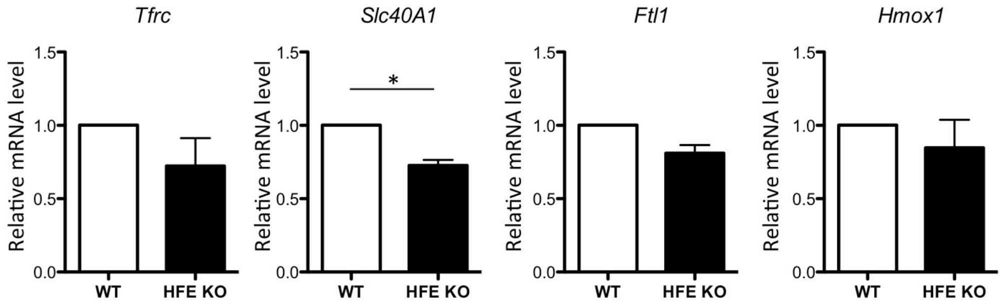

Supplementary Figure 5: Iron-related genes in AM isolated from the lung of WT and HFE KO mice:

Tfrc, *Slc40A1*, *Ftl1* and *Hmox1* mRNA levels relative to *Ppia* analyzed by real-time PCR in AM. The WT AM cells have been used as reference for each single experiment of AM isolation performed on an-age matched couple of WT and HFE KO mice, and the data that are expressed in fold change. Error bars represent SEM for n=3 mice in each group. Statistical significance is indicated by asterisks (*p< 0.05).

Figure S6

A

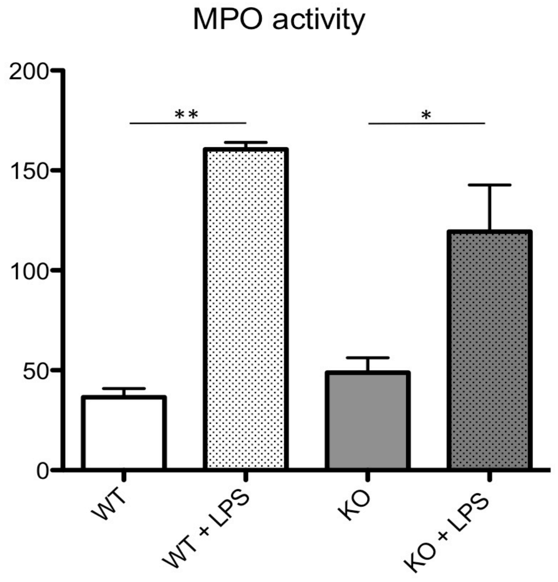

B

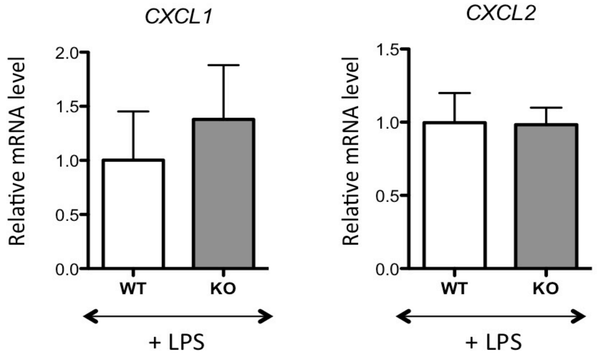

Supplementary Figure 6: MPO activity and chemokine responses in the lung of IP LPS treated WT and Hepc KO mice:

MPO activity, expressed in units per mg of tissue was measured in the lung of mice IP injected for 6h by LPS (A). Error bars represent SEM for n=3-6 mice by group. Statistical analysis was performed using one-way analysis of variance (ANOVA), Bonferroni's multiple comparison. Statistical significance is indicated by * asterisks (*$p< 0.05$, **$p< 0.001$).

CXCL1 and *CXCL2* mRNA levels relative to *Ppia*, analyzed by real-time PCR in the lung of mice after 5h of intranasal LPS instillation (B). Changes are expressed relative to WT injected mice.

Figure S7

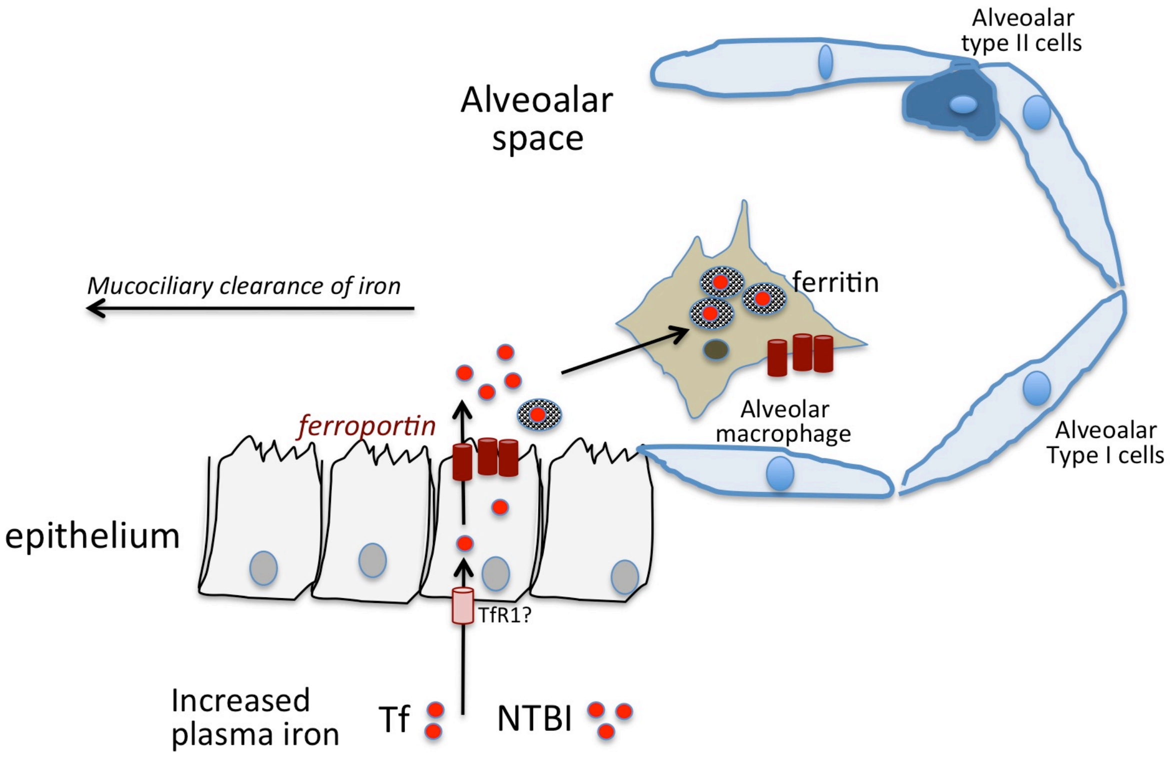

Supplementary Figure 7: Schematic representation of iron handling in the lung of the Hepc KO mice (Tf , transferrin, NTBI, non transferrin bouf iron).
